# Evaluation of specific RBE in different cells of hippocampus under high-dose proton irradiation in rats

**DOI:** 10.1038/s41598-024-58831-z

**Published:** 2024-04-08

**Authors:** Shengying Zhou, Xingchen Ding, Yiyuan Zhang, Yuanyuan Liu, Xiaowen Wang, Yujiao Guo, Jianguang Zhang, Xiao Liu, Guanzhong Gong, Ya Su, Lizhen Wang, Miaoqing Zhao, Man Hu

**Affiliations:** 1 School of Clinical Medicine, Shandong Second Medical University, Weifang, 261053 Shandong China; 2 Department of Radiation Oncology, Shandong Cancer Hospital and Institute, Shandong First Medical University and Shandong Academy of Medical Sciences, NO.440 Ji Yan Road, Jinan, 250117 Shandong China; 3 Department of Pathology, Shandong Cancer Hospital and Institute, Shandong First Medical University and Shandong Academy of Medical Sciences, Jinan, 250117 Shandong China; 4 Shandong University cancer center, Jinan, 250100 Shandong China; 5 Affiliated Hospital of Jining Medical College, Jining, 272067 Shandong China; 6 Zibo Wanjie Cancer Hospital, Zibo, 255202 Shandong China; 7 960 Hospital of the Joint Logistics Support Force of the Chinese People’s Liberation Army, Jinan, 250031 Shandong China

**Keywords:** Relative biological effectiveness, Proton beam therapy, Hippocampus, Neuronal damage, Radiation-induced brain injury, Radiotherapy, Radiotherapy

## Abstract

The study aimed to determine the specific relative biological effectiveness (RBE) of various cells in the hippocampus following proton irradiation. Sixty Sprague–Dawley rats were randomly allocated to 5 groups receiving 20 or 30 Gy of proton or photon irradiation. Pathomorphological neuronal damage in the hippocampus was assessed using Hematoxylin–eosin (HE) staining. The expression level of NeuN, Nestin, Caspase-3, Olig2, CD68 and CD45 were determined by immunohistochemistry (IHC). The RBE range established by comparing the effects of proton and photon irradiation at equivalent biological outcomes. Proton_20Gy_ induced more severe damage to neurons than photon_20Gy_, but showed no difference compared to photon_30Gy_. The RBE of neuron was determined to be 1.65. Similarly, both proton_20Gy_ and proton_30Gy_ resulted in more inhibition of oligodendrocytes and activation of microglia in the hippocampal regions than photon_20Gy_ and photon_30Gy_. However, the expression of Olig2 was higher and CD68 was lower in the proton_20Gy_ group than in the photon_30Gy_ group. The RBE of oligodendrocyte and microglia was estimated to be between 1.1 to 1.65. For neural stem cells (NSCs) and immune cells, there were no significant difference in the expression of Nestin and CD45 between proton and photon irradiation (both 20 and 30 Gy). Therefore, the RBE for NSCs and immune cell was determined to be 1.1. These findings highlight the varying RBE values of different cells in the hippocampus in vivo. Moreover, the actual RBE of the hippocampus may be higher than 1.1, suggesting that using as RBE value of 1.1 in clinical practice may underestimate the toxicities induced by proton radiation.

## Introduction

Malignant brain tumors mainly include primary and secondary types, both of which pose a significant threat to patient lives and have poor prognoses. Radiotherapy (RT) has been shown to be effective in improving the overall survival (OS) and local control rate (LCR) of patients with cerebral malignancies^1,2^. Proton beam therapy (PBT) is one of the most advanced RT modalities available globally, leveraging a unique physics advantage known as the Bragg peak. This feature is considered to offer a more precise tumor control dose and lower treatment-related toxicity than photon therapy^3–5^. Currently, the prescribed dose of protons used in clinical applications is typically determined by a constant relative biological effectiveness (RBE) of 1:1^6,7^. RBE is defined as the ratio of photon versus proton dose when producing the same biological effect^8^. However, the RBE of proton (1.1) was derived from cell proliferation experiments conducted by previous researchers, and further in vivo experiments are needed to validate these findings.

At present, a growing number of studies have found that proton RBE is variable. Solely relying on a fixed value of 1.1 may lead to seriously underestimation of the effect of protons on normal tissue damage, potentially resulting in a higher incidence and severity of radiotoxicity, particularly in organs such as heart and lungs^9,10^. Similarly, radiation-induced brain injury (RIBI) has also been observed clinically after proton therapy^11,12^. RIBI includes not only acute adverse effects such as headache, dizziness, nausea and drowsiness^13^, but also delayed brain injury, characterized by hippocampal-related learning and cognitive impairment and brain necrosis which even can eventually lead to uncontrolled brain herniation and death^14^. Prezado et al. found that radio-necrosis of brain tissue could be induced when rats were irradiated with protons (25 Gy or more)^15^. Williams et al. found that rats exposed to 14 and 17 Gy proton irradiation were defective in the Morris water maze task test, which reflected hippocampal learning and memory function^16^. Lawrence et al. concluded that the five-year risk of RIBI with normally fractionated RT at total doses of 72 Gy was ~ 5%^17^. The hippocampus is highly sensitive to radiation and is one of the most important organs at risk (OAR) in cranial RT^18^. It had been demonstrated that that exposure to high linear energy transfer (LET) irradiation caused short- and long-term flaws in hippocampus-dependent cognition and learning^19^,and could also lead to severe dysfunction of the central nervous system (CNS)^20^. Therefore, it is very crucial to protect brain tissues, especially the hippocampal region, during RT.

Various factors can influence the variation of proton RBE, such as the radiation dose/fraction, α/β, LET, and biological end points^6^. The dependence of RBE on tissue-specific α/β ratio is well-established^21^. Previous studies have indicated that the neurological tissues typically have a lower α/β ratio^22^, and tissues with low α/β values show higher RBE values than those with high α/β values^23^. Due to the heterogeneity of the brain, irradiation may have different biological effects in different brain areas. Despite great efforts have been taken to estimate clinical RBE using mathematical models^7^, the true RBE of normal brain tissues remains unknown. Additionally, as protons decelerate, LET increases with depth, potentially leading to higher RBE in normal tissue below the target area^24^. Given the anatomical location of the hippocampus beneath tumor targets and its composition of neurons, oligodendrocytes, microglia and neural stem cells (NSCs) in the subgranular zone (SGZ) of dentate gyrus (DG), it is speculated that the RBE of the hippocampus may be higher than 1.1^25^. In vitro experiments confirmed the RBE of ~ 1.35 for glioma U87 when LET = 2.6^26^. Clonogenic assay showed that the RBE of skin fibroblasts between 1.4 and 2.2 when LET of 2 to 2.6^26,27^. To date, there have been no relevant studies have explored the actual RBE of the hippocampus by comparing the damage differences in different cells caused by proton versus photon irradiation.

Therefore, this study aims to investigate the difference of biological effects of different cells in hippocampus after proton and photon irradiation by using a rat model of RIBI, and subsequently calculate the RBE for different cells. This will be crucial for accurately determining the RBE values of brain tissue, thereby significantly advancing the clinical application of PBT in malignant brain tumors and promoting the dawn of an era of precise proton therapy for malignant brain tumors.

## Materials and methods

### Rats and irradiation procedures

Sixty healthy male rats (6-week-old, Sprague–Dawley (SD), 200–220 g, from Jinan Pengyue Laboratory Animal Breeding Co. Ltd.) were housed in a sterile animal house under a 12:12-h light–dark cycle, with free access to water and food. Ethics statement: All animal experiments were conducted in accordance with the animal welfare and ethical guidelines and approval was granted by the Ethics Committee of Shandong Cancer Hospital (ID: 201911022). And the study was carried out in accordance with ARRIVE guidelines.

A rat model of acute RIBI needed to be established in this study, therefore, two doses of 20 Gy and 30 Gy were selected for whole brain radiation in rats (proton dose was converted to photon dose according to 1:1.1). Rats were randomly grouped as follows (n = 12): proton_20_ (20 Gy, RBE), proton_30_ (30 Gy, RBE), photon_20_ (20 Gy), photon_30_ (30 Gy) and control.

Proton irradiation was performed using a proton radiation accelerator (PROTEUS235 proton therapy system from IBA, Belgium). The anesthetized rats were fixed on the radiation table and exposed to the radiation area, using the intersection of the median line of the parietal bone of the skull and the posterior line of the two ears of the rats as the irradiation localization center, the axial distance of the source was adjusted to be 230 cm, and the proton beams had an energy of 230 MeV and a dose rate of 2 Gy/min, the depth and width of the SOBP were measured by the water tank before exposure, and 3 cm of SOBP was obtained by the range modulator to ensure the uniformity of the radiation dose to the whole brain of the rats. Detailed irradiation parameters can be found as Supplementary Table S1 online.

Photon irradiation was performed by using a photon linear accelerator (Valerian VitalBeam, USA) with a dose rate of 1400 MU/min and an energy of 6 MV, adjusting the source skin distance to 100 cm and covering the head with a 1-cm tissue-equivalent membrane. All rats were anesthetized with isoflurane (2.5% in air) before irradiation.

### Follow-up processing

The rats were anesthetized and euthanized by intraperitoneal injection of 50 mg/kg pentobarbital sodium. Half of all groups’ rats were sacrificed at 24 h and the other half rats were sacrificed at 7 days post-irradiation. Brain fixation was performed by trans-cardiac perfusion with heparin-containing 0.9% saline followed by a rapid drip of 4% paraformaldehyde. Then the brains were removed, and the 3-mm-thick blocks of tissue in coronal section from the hippocampal region were immersed in 10% formalin for 24 h. The pathological tissue wax blocks were prepared by paraffin embedding, and the consecutive coronal sections were made with a thickness of 3 um. Hematoxylin–eosin staining (HE staining) was performed, scanned using Zeiss digital slice scanner and observed by ZEN (BLUE) 2012 software, and two pathologists were asked to (double-blind) analyze and count the damaged neurons in each subregion of the hippocampus in the same coronal plane of pathology sections, with four random fields of view selected for each subregion, then the ratio of damaged neurons to all the neurons under the field of view was calculated. Damaged neurons included dark neurons, necrotic neurons and apoptotic neurons, which were cytomorphologically defined as eosinostaining enhanced, cytoplasmic looseness, nucleolar segregation, shrunken and deformed cytoplasm, hyperchromatic nucleus, karyopyknosis and karyolysis. Immunohistochemistry (IHC) analysis was performed to assess the expression of NeuN (Abcam, dilution: 1:200), Nestin (ABclonal, dilution: 1:100), Caspase-3 (Proteintech, dilution: 1:300), Olig2 (Venus gene, dilution: 1:200), CD68 (Venus gene, dilution: 1:100) and CD45 (Venus gene, dilution: 1:500) in the brain. Average optical density (AOD) was performed using ImageJ software version 1.54 (NIH, Bethesda, MD) to assess the density of positive cells.

RBE was defined as the ratio of the photon and proton dose when these two irradiation types produce equivalent biology output. Consider that different cells in the hippocampus have different effects after irradiation, we determined different biological output of them. The specific algorithm of the RBE range in different types of cells were showed in Table 1.

**Table 1:** Relative biological effectiveness (RBE) calculated in different situations.

Proton dose (RBE) vs. photon dose	Biological effects of proton vs. biological effects of photon	*p* value	RBE = photon dose/proton dose (actual)
20 Gy vs. 20 Gy or 30 Gy vs. 30 Gy	>	≤ 0.05	> 1.1
	>	> 0.05	= 1.1
	<	≤ 0.05	< 1.1
	<	> 0.05	= 1.1
20 Gy vs. 30 Gy	>	≤ 0.05	> 1.65
	>	> 0.05	= 1.65
	<	≤ 0.05	< 1.65
	<	> 0.05	= 1.65

### Statistics

After normality testing (Shapiro–Wilk test), the differences between the three groups were analyzed using one-way Analysis of Variance (ANOVA) followed by Fisher’s least significant difference (LSD) post-hoc test. Two-sided unpaired Student’s t-test or Mann–Whitney U test was used for comparison of two groups. Results were considered statistically significant when *p* < 0.05. Data are presented as mean ± the standard error of the mean (SEM). All statistical analyses were performed using SPSS v26.0 (IBM, Chicago, Illinois, USA) or GraphPad Prism Software v9.5 (GraphPad, San Diego, California, USA).

### Ethics approval

This study was performed in line with the principles of the Declaration of Helsinki. And the study is reported in accordance with ARRIVE guidelines. Approval was granted by the Ethics Committee of Shandong Cancer Hospital (No: 201911022).

## Results

### Differences in damage of neurons caused by proton versus photon irradiation

**Figure 1:**
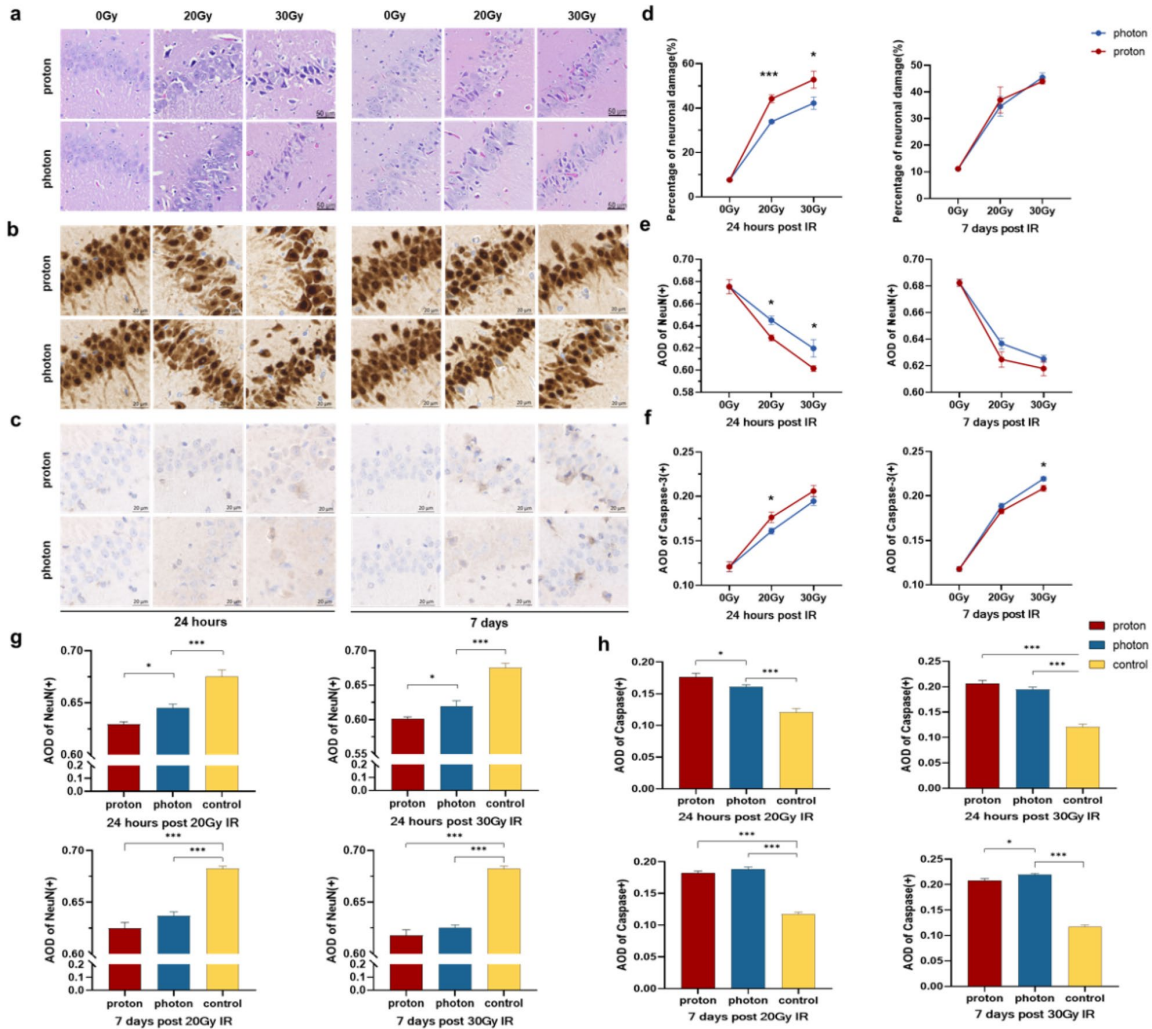
The effects of proton and photon irradiation (IR) on hippocampal neurons. (**a**) Differences in neuronal damage caused by proton and photon IR at different doses and time points assessed through HE staining. (**b**) IHC staining shows differences in NeuN expression between proton and photon IR. (**c**) IHC staining shows differences in Caspase-3 expression between proton and photon IR. (**d**) The neuronal damage rate post-irradiation is dose-dependent. (**e**) The expression of NeuN post-irradiation is dose-dependent. (**f**) The expression of Caspase-3 post-irradiation is dose-dependent. (**g**) Differences in average optical density (AOD) values of NeuN among groups under different time and dosage conditions. (**h**) Differences in AOD values of Caspase-3 among groups under different time and dosage conditions. *p < 0.05; **p < 0.01; ***p < 0.001.

In the early phase of acute injury (24 h after irradiation), irregular arrangement of hippocampal neurons, loose cytoplasm, shrunken and deformed cytoplasm, hyperchromatic nucleus, karyopyknosis, karyorrhexis and karyolysis could be observed in the HE-stained sections of both the proton and photon groups compared with the control group (Fig. 1a). The degree of injury was found to be dose-dependent (Fig. 1d).

Protons caused more severe neuronal damage than photons at 24 h post-irradiation, no matter the dose was 20 or 30 Gy. HE staining results showed that the overall neuronal damage rate was approximately 10% higher in the proton group than in the photon group (20 Gy:* p* < 0.001, 30 Gy: *p* = 0.047). Subsequently, neuronal damage was assessed in each hippocampal subregion (Supplementary Fig. S1 online). The results showed that a significantly higher rate of neuronal damage in the proton group compared to the photon group across CA1, CA2, CA3, and CA4 regions (Table 2). While there was a difference in neuronal damage in the DG region, it did not reach statistical significance (Table 2). Similarly, IHC analysis using NeuN and Caspase-3 markers further supported that both the degree of neuronal damage and the level of apoptosis were more severe in the proton group than that in the photon group (NeuN: *p* = 0.027, *p* = 0.049, Fig. 1b,g, Tables 2; Caspase-3: *p* = 0.048, *p* = 0.162, Fig. 1c,h, Tables 2). The expression extent of neurons and the level of apoptosis demonstrated by IHC after irradiation also show a dose-dependent relationship (Fig. 1e,f).

**Table 2:** The biological effects of different indicators at various time points and dosage levels.

		24 h after 20 Gy IR			24 h after 30 Gy IR		
		Proton	Photon	*p*	Proton	Photon	*p*
HE	CA1	33.64% ± 2.31%	26.25% ± 1.78%	0.009	41.81% ± 5.62%	32.69% ± 3.61%	0.118
	CA2	44.31% ± 2.72%	34.35% ± 3.80%	0.173	55.12% ± 2.94%	40.54% ± 4.00%	0.048
	CA3	52.69% ± 1.68%	42.04% ± 2.61%	0.001	59.35% ± 3.40%	44.16% ± 3.35%	0.029
	CA4	48.75 ± 4.46%	35.30% ± 1.50%	0.003	65.37% ± 5.30%	56.96% ± 3.70%	0.536
	DG	41.84% ± 4.41%	31.73% ± 1.34%	0.199	42.51% ± 5.11%	36.76% ± 3.50%	0.277
IHC	NeuN	0.629 ± 0.003	0.645 ± 0.004	0.027	0.602 ± 0.003	0.620 ± 0.008	0.049
	Caspase-3	0.176 ± 0.006	0.161 ± 0.003	0.048	0.206 ± 0.006	0.195 ± 0.005	0.162

		7 days after 20 Gy IR			7 days after 30 Gy IR		
		Proton	Photon	*p*	Proton	Photon	*p*
HE	CA1	20.01% ± 2.08%	20.06% ± 1.78%	0.984	28.71% ± 2.63%	31.37% ± 2.62%	0.867
	CA2	37.43% ± 5.15%	34.61% ± 5.28%	0.976	49.16% ± 2.63%	52.45% ± 3.08%	0.821
	CA3	39.71% ± 7.78%	38.62% ± 4.75%	0.999	50.29% ± 4.81%	53.00% ± 2.44%	0.949
	CA4	47.10% ± 6.78%	43.37% ± 8.44%	0.982	47.10% ± 2.27%	46.37% ± 3.92%	0.998
	DG	40.72% ± 7.91%	36.31% ± 5.71%	0.961	44.57% ± 2.78%	44.15% ± 3.94%	1.0
IHC	NeuN	0.625 ± 0.006	0.637 ± 0.004	0.069	0.618 ± 0.005	0.625 ± 0.003	0.602
	Caspase-3	0.183 ± 0.003	0.188 ± 0.003	0.194	0.209 ± 0.003	0.219 ± 0.002	0.016

At the late phase of acute injury (7 days after irradiation), HE staining revealed no significant differences in the rate of neuronal damage between proton group and the photon group in each subregion (Table 2). Similarly, there was no significant difference in the expression of NeuN between the two groups (20 Gy: *p* = 0.069, 30 Gy: *p* = 0.602, Fig. 1g). While there was no significant difference between the groups after 20 Gy irradiation (*p* = 0.194, Fig. 1h), the expression of Caspase-3 was lower in the proton group compared to the photon group after 30 Gy irradiation (*p* = 0.016, Fig. 1h).

### Differences in the effects of proton versus photon irradiation on NSCs

**Figure 2:**
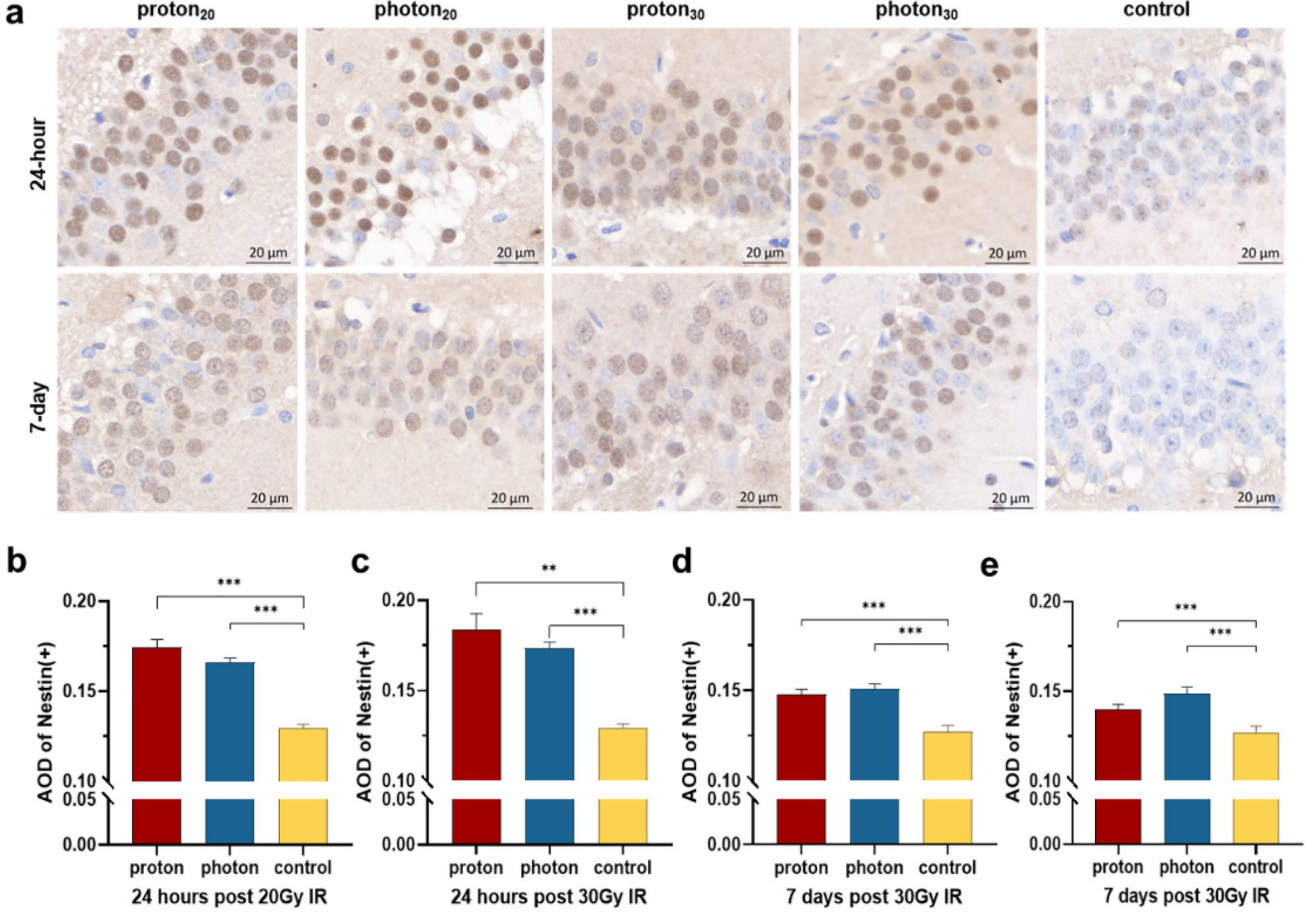
Changes in the expression of NSCs in the brain after IR. (**a**) IHC shows the expression of Nestin under different types of IR at different times. (**b**) The AOD values of Nestin(+) at 24 h after 20 Gy IR. (**c**) The AOD values of Nestin(+) at 24 h after 30 Gy IR. (**d**) The AOD values of Nestin(+) at 7 days after 20 Gy IR. (**e**) The AOD values of Nestin(+) at 7 days after 30 Gy IR. *p < 0.05; **p < 0.01; ***p < 0.001.

IHC analysis was utilized to detect Nestin-positive cells and evaluate the effects of proton versus photon irradiation on hippocampal NSCs (Fig. 2a). The results indicated that irradiation could up-regulate the expression levels of Nestin, with significant differences observed between the proton or photon groups and the control group (all *p* < 0.001). After 24 h post-irradiation, the expression of Nestin in the proton group was slightly higher than that in photon group, although this difference was not statistically significant (20 Gy: 0.174 ± 0.004 vs. 0.166 ± 0.002, *p* = 0.081, Fig. 2b; 30 Gy: 0.184 ± 0.009 vs. 0.173 ± 0.004, *p* = 0.637, Fig. 2c). This suggests that proton-induced activation of hippocampal NSCs occurs more rapidly in the initial phase of injury than that induced by photons. After 7 days, the Nestin immunoreactivity in the proton group became slightly weaker than in the photon group (20 Gy: 0.148 ± 0.003 vs. 0.151 ± 0.002, *p* = 0.370, Fig. 2d; 30 Gy: 0.140 ± 0.003 vs. 0.149 ± 0.004, *p* = 0.075, Fig. 2e).

### Differences in the effects of proton versus photon irradiation on glial cells

**Figure 3:**
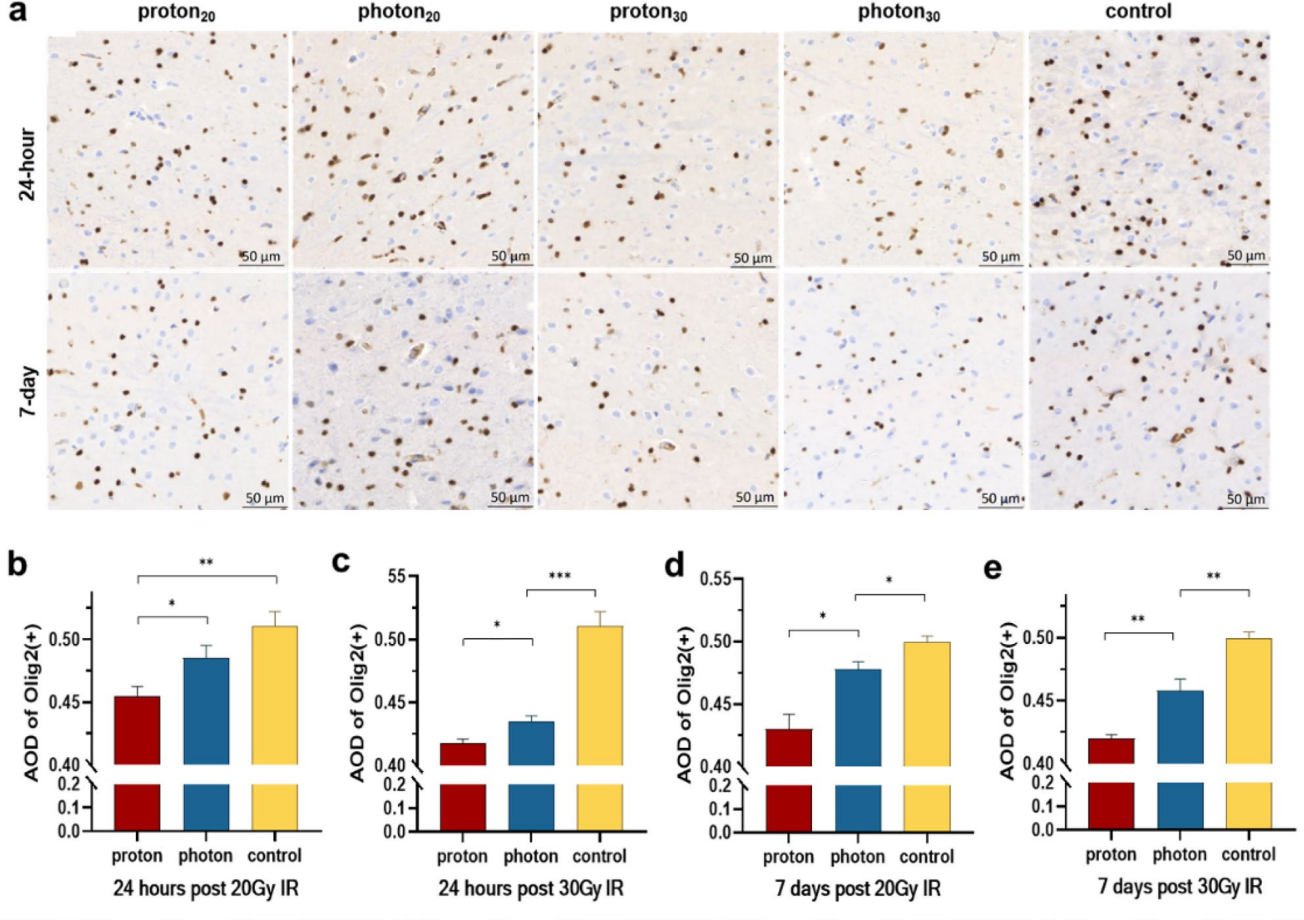
Changes in the expression of oligodendrocytes in the brain after IR. (**a**) IHC shows the expression of olig2 under different types of IR at different times. (**b**) The AOD values of olig2(+) at 24 h after 20 Gy IR. (**c**) The AOD values of olig2(+) at 24 h after 30 Gy IR. (**d**) The AOD values of olig2(+) at 7 days after 20 Gy IR. (**e**) The AOD values of olig2(+) at 7 days after 30 Gy IR. *p < 0.05; **p < 0.01; ***p < 0.001.

Protons significantly reduced the number of oligodendrocytes in the hippocampus compared to photons (Fig. 3a). IHC analysis showed that the AOD of Olig2 in the proton group was significantly lower than in the photon group (20 Gy: 0.455 ± 0.007 vs. 0.485 ± 0.010, *p* = 0.044, Fig. 3b; 30 Gy: 0.418 ± 0.003 vs. 0.435 ± 0.005, *p* = 0.045, Fig. 3c), and this differences still observable even at 7 days post-irradiation (20 Gy: 0.430 ± 0.012 vs. 0.478 ± 0.006, *p* = 0.022; Fig. 3d; 30 Gy: 0.420 ± 0.003 vs. 0.458 ± 0.009, *p* = 0.001, Fig. 3e).

**Figure 4:**
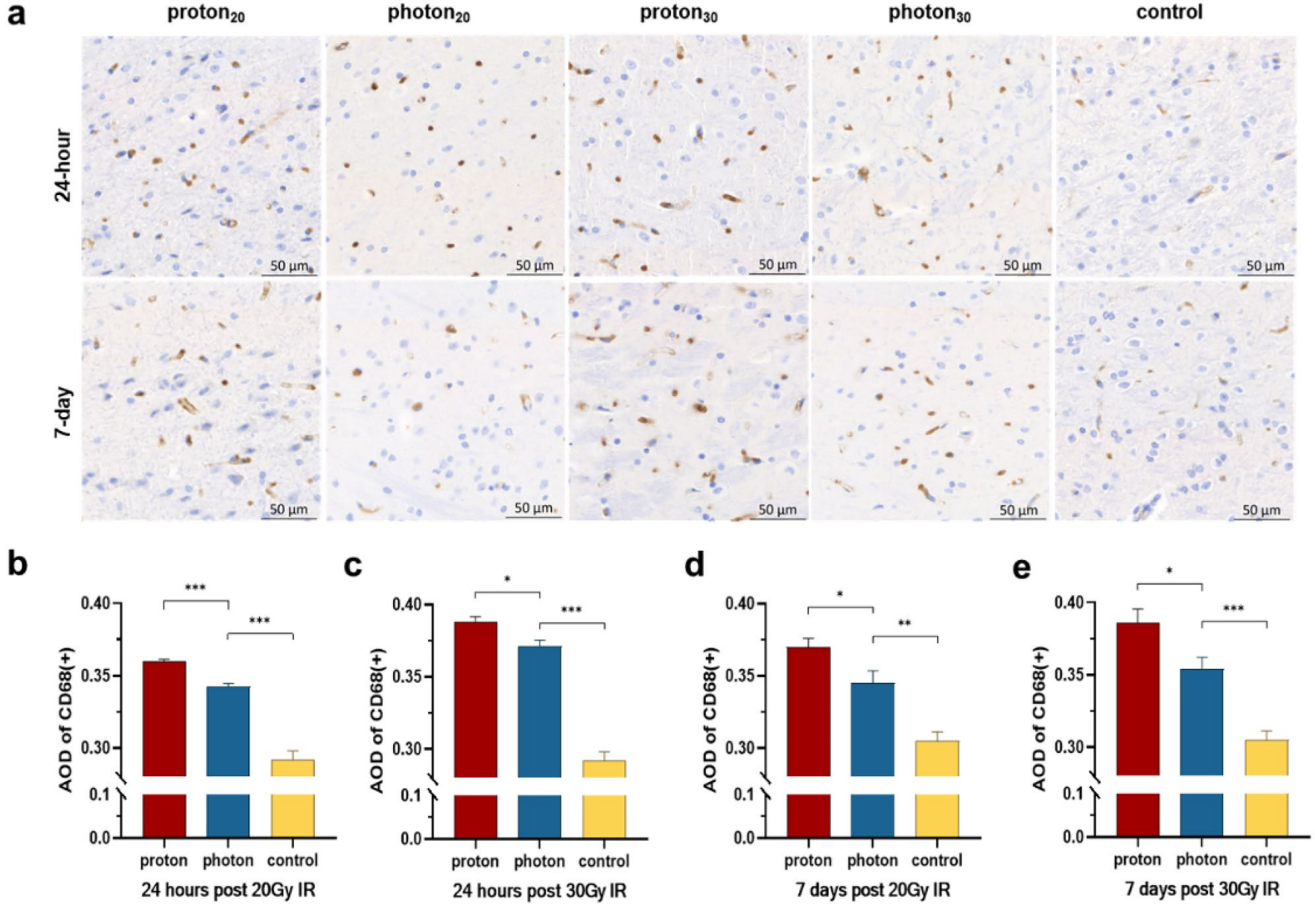
Changes in the expression of activated microglia in the brain after IR. (**a**) IHC shows the expression of CD68 under different types of IR at different times. (**b**) The AOD values of CD68(+) at 24 h after 20 Gy IR. (**c**) The AOD values of CD68(+) at 24 h after 30 Gy IR. (**d**) The AOD values of CD68(+) at 7 days after 20 Gy IR. (**e**) The AOD values of CD68(+) at 7 days after 30 Gy IR. *p < 0.05; **p < 0.01; ***p < 0.001.

However, the changes in microglia after irradiation were opposite to those of oligodendrocytes. Proton irradiation significantly increased the number of activated microglia compared to photon irradiation (Fig. 4a). Whether at 24 h or 7 days after irradiation, the expression of CD68 in hippocampus was higher in the proton-treated group than in the photon-treated group (24 h: 20 Gy: 0.360 ± 0.001 vs. 0.343 ± 0.002, *p* < 0.001, Fig. 4b; 30 Gy: 0.388 ± 0.003 vs. 0.371 ± 0.004, *p* = 0.022, Fig. 4c; 7d: 20 Gy: 0.370 ± 0.006 vs. 0.345 ± 0.008, *p* = 0.022, Fig. 4d; 30 Gy: 0.386 ± 0.010 vs. 0.355 ± 0.008, *p* = 0.014; Fig. 4e). In summary, proton therapy appears to induce a greater activation of microglia compared to photon therapy.

### Differences between photon and photon irradiation in influencing immune cell aggregation

**Figure 5:**
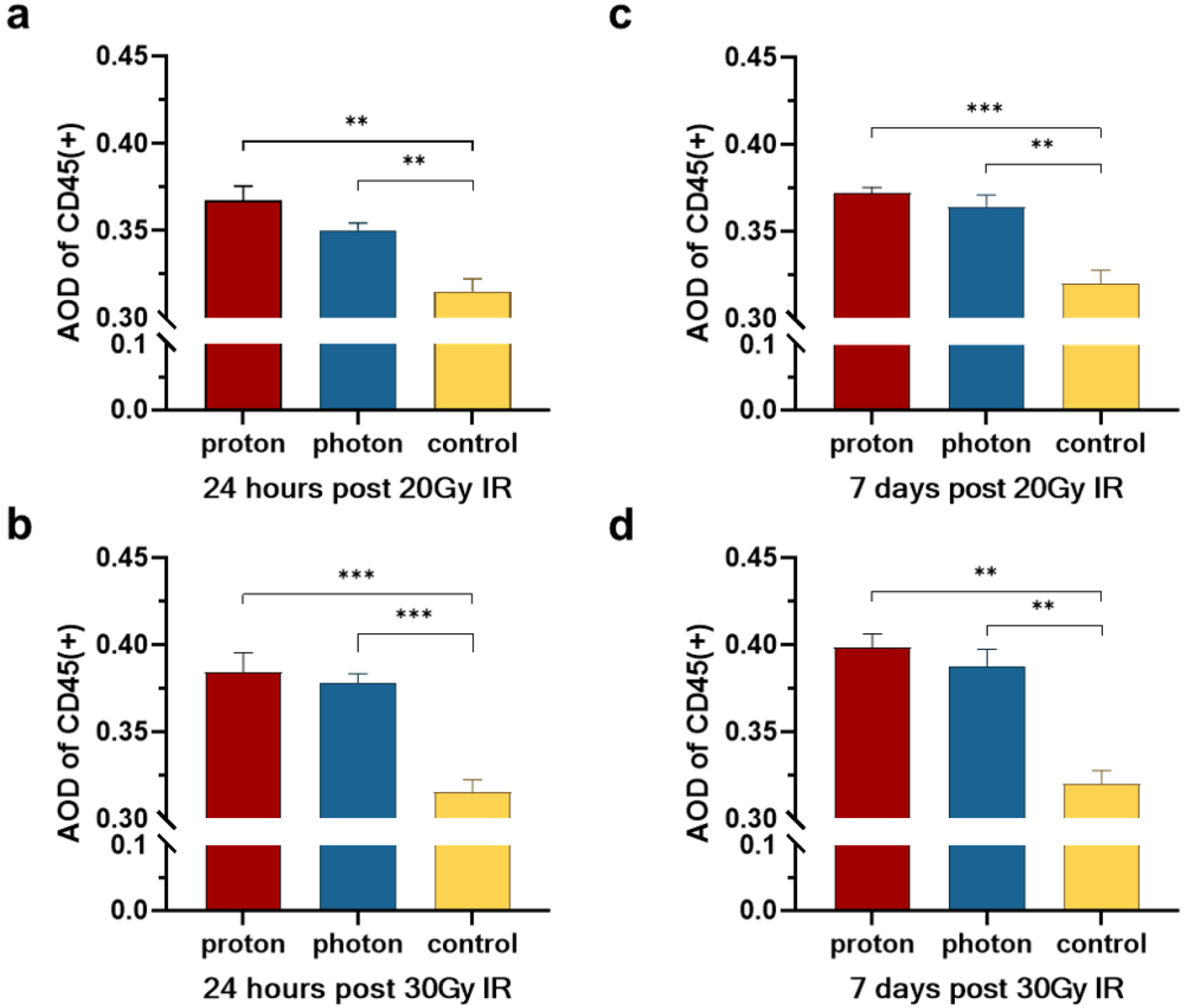
Changes in the expression of immune cells in the brain after IR. (**a**) The AOD values of CD45(+) at 24 h after 20 Gy IR. (**b**) The AOD values of CD45(+) at 24 h after 30 Gy IR. (**c**) The AOD values of CD45(+) at 7 days after 20 Gy IR. (**d**) The AOD values of CD45(+) at 7 days after 30 Gy IR. *p < 0.05; **p < 0.01; ***p < 0.001.

The results of IHC analysis revealed that both proton and photon radiation exhibited similar levels of CD45 expression in 24 h after exposure, regardless of whether in the 20 Gy or the 30 Gy group (20 Gy: 0.367 ± 0.008 vs. 0.350 ± 0.004, *p* = 0.098, Fig. 5a; 30 Gy: 0.385 ± 0.011 vs. 0.379 ± 0.005, *p* = 0.607, Fig. 5b). One week after irradiation, CD45 expression in both groups remained consistent on the 7th day (20 Gy: 0.372 ± 0.003 vs. 0.364 ± 0.007, *p* = 0.381, Fig. 5c; 30 Gy: 0.399 ± 0.008 vs. 0.388 ± 0.010,* p* = 0.371, Fig. 5d). In conclusion, regardless of dosage or timing, the results demonstrate no significant differences in the impacts of proton and photon radiation on immune cells.

### Real RBE values of different cells in the hippocampus after proton irradiation

Over time, multiple confounders such as immune cells and inflammatory factors have been involved in the effects of brain tissue damage. It is now recognized that calculating RBE solely based on a single biological endpoint is no longer feasible. Therefore, the RBE values was calculated in various cell types during the early stage of acute injury (24 h post-irradiation, Table 3). The results showed that proton-induced neuronal damage was more severe compared to photon-induced damage when assessing biological equivalent doses (proton_20_ vs. photon_20_: *p* < 0.001, proton_30_ vs. photon_30_: *p* = 0.047). However, there was no significant difference between the proton_20_ group and the photon_30_ group (proton_20_ vs. photon_30_: *p* = 0.550), reaching an equivalent biological end point. Consequently, the actual RBE of neuron cells was calculated to be 1.65 according 30 Gy/ (20/1.1) Gy. Applying the same method, it was observed that protons had a greater impact on oligodendrocytes and microglia compared to photons under equivalent biological dose irradiation (oligodendrocyte: *p* = 0.044 and *p* = 0.045; microglia: *p* < 0.001 and *p* = 0.022). Nonetheless, when comparing the proton_20_ with photon_30_ group, the biological effect of the photon_30_ group was still higher and did not reach the equivalent biological endpoint (oligodendrocyte: *p* = 0.040; microglia: *p* = 0.048). Therefore, the actual RBEs of both oligodendrocytes and microglia were estimated to be between 1.1 and 1.65. In contrast, for NSCs and immune cells, both proton and photon irradiation groups reached effective biological endpoints at equivalent biological doses. Thus, the actual RBEs of NSCs and immune cells were suggested to be around 1.1.

**Table 3:** Different cells in the hippocampus have different RBE.

Cell type	proton		photon		*p* value
	dose (RBE)	biological effects	dose	biological effects	
Neuron	20	44.25% ± 1.79%	20	33.94% ± 0.71%	< 0.001
	30	52.83% ± 3.80%	30	42.22% ± 2.74%	0.047
	20	44.25% ± 1.79%	30	42.22% ± 2.74%	0.550
	RBE = 1.65				
NSC	20	0.174 ± 0.004	20	0.166 ± 0.002	0.081
	30	0.184 ± 0.009	30	0.173 ± 0.004	0.637
	20	0.174 ± 0.004	30	0.173 ± 0.004	0.864
	RBE = 1.1				
Oligodendrocyte	20	0.455 ± 0.007	20	0.485 ± 0.010	0.044
	30	0.418 ± 0.003	30	0.435 ± 0.005	0.045
	20	0.455 ± 0.007	30	0.435 ± 0.005	0.040
	1.1 < RBE < 1.65				
Microglia	20	0.360 ± 0.001	20	0.343 ± 0.002	< 0.001
	30	0.388 ± 0.003	30	0.371 ± 0.004	0.022
	20	0.360 ± 0.001	30	0.371 ± 0.004	0.048
	1.1 < RBE < 1.65				
Immune cell	20	0.367 ± 0.008	20	0.350 ± 0.004	0.098
	30	0.385 ± 0.011	30	0.379 ± 0.005	0.607
	20	0.367 ± 0.008	30	0.379 ± 0.005	0.273
	RBE = 1.1				

NSC is neural stem cell.

## Discussion

Our results showed that 24 h post-irradiation, neuronal damage was more severe in terms of pathological morphology in the proton group compared to the photon group (20 Gy:* p* < 0.001, 30 Gy: *p* = 0.047). Protons exhibited stronger abilities to inhibit neurons and promote cell apoptosis than photons (20 Gy: *p* = 0.027 and *p* = 0.048), with a RBE 1.65 for neuronal cells. These results indicate that protons cause more severe damage to neurons than photons at equivalent biological doses of radiation. At present, most of related studies focus on the differences in tumor tissues and cells following proton and photon irradiation, limited studies have focused on the impact on normal tissues after irradiation. For instance, Choi et al. observed that irradiation of normal intestinal tissues in mice with 6-MV photons or 230-MeV protons resulted in higher apoptotic cell death in the jejunum with proton irradiation (*p* < 0.001), indicating increased gastrointestinal toxicity^28^. Furthermore, studies specifically addressing the hippocampus are scarce. Parihar et al. found that exposure to proton irradiation led to more pronounced disruptions in dendritic morphology compared to photon irradiation^29^. Howe and Kiffer et al. demonstrated that radiation significantly reduced mushroom spine density in the hippocampal CA1, CA3 and DG regions, suggesting detrimental effects on mature neurons associated with learning and memory in the hippocampus^30^. These findings are in agreement with ours. From the molecular mechanism perspective, the difference in cell killing between protons and photons may be more intricate^7^. The amount of activated γ-H2AX can be used to assess the amount of DNA damage^31^. Studies have shown contrasting results, with Gerelchuluun et al. demonstrating a significantly increase in γ-H2AX levels in human tumor cell lines ONS76 and MOLT4, 30 min after 2 Gy irradiation with 200 MeV of proton compared with 10 MV of X-ray irradiation (ONS76: *p* = 0.006; MOLT4: *p* = 0.025)^32^. Conversely, Dokic et al. found that the number of γ-H2AX positive nuclei in the brains of healthy C57BL/6 mice was significantly reduced at 7 days compared with 1 h after proton irradiation, indicating DNA damage repair^33^. Lohberger et al. also suggested that proton irradiation activated DNA repair mechanisms more effective than photon did^34^. In this study, no significant difference in pathomorphology of neurons and the expression of NeuN in IHC at 7 days after irradiation between proton and photon groups (*p* > 0.05), which indicates that the mechanism of DNA damage repair after proton irradiation is not the same as that of photon, resulting in different rates of DNA repair. Besides, ionizing radiation also leads to the production of reactive oxygen species (ROS) that can damage DNA indirectly by reacting with molecular oxygen to form stable DNA peroxides^31^. Giedzinksi et al. observed that elevated ROS levels in rat hippocampal neural precursor cells after exposure to protons near the Bragg peak at 250 MeV compared to X-rays (*p* < 0.05)^35^. In summary, we propose that the RBE of neurons is likely higher than 1.1. Therefore, in PBT for brain tumors, minimizing radiation exposure to hippocampal neurons while ensuring therapeutic efficacy is crucial.

It should be noted that the effects of proton radiation on NSCs are different from those of neurons. Radiation may stimulate the activation and redifferentiation of NSCs, which mainly located in the subventricular zone (SVZ) and the SGZ in the adult brain. NSCs are highly sensitive to ionizing radiation and have the ability to differentiate into neurons, oligodendrocytes and microglia^36^. Nestin protein, also known as neuroepithelial stem cell protein, is an embryonic intermediate filament protein abundantly presented in the proliferative zone of the CNS^37^. While Nestin is minimally expressed in mature neurons and glial cells, its levels increase in damaged neural tissue^38,39^. IHC analysis revealed that proton radiation led to a higher expression of Nestin compared to photon radiation 24 h post-irradiation, although the difference was not statistically significant (*p* > 0.05). Therefore, the RBE for NSCs is 1.1. The elevated expression of Nestin reflects the activated state of NSCs, and its proteins, may protect the nervous system from injury by promoting cell proliferation, differentiation, and neuroglial scar formation, as well as promoting the formation of new synapses in neurons, represents neurogenesis or neuroremodeling^40^. This suggests that proton may be more effective than photon in promoting NSCs differentiation at early phase after irradiation. It is plausible that this mechanism contributes to the diminishing disparity in neuronal damage between protons and photons beyond 7 days post-irradiation.

The biological effects of proton and photon irradiation are also different for other types of cells in brain tissue. Olig2, a key factor in oligodendrocyte transcription^41^, is specifically expressed in normal brain oligodendrocytes. Our study observed a significant decrease in Olig2 expression in brain tissue following proton irradiation compared to photon irradiation (20 Gy: *p* = 0.044; 30 Gy: *p* = 0.045). As a result, the RBE of oligodendrocytes ranged from 1.1 to 1.65, indicating a stronger inhibitory effect of protons on oligodendrocytes compared to photons. The reduction of oligodendrocyte, which is required for myelination, eventually leads to demyelination of brain neurons and necrosis of the white matter^42^. Therefore, a reduction in oligodendrocyte leads to further neuronal damage and a decrease in the overall number of neurons.

Previous studies have shown that the increase in inflammation-associated cytokines (such as IL-6, IL-1β, TNF-α and so on) after RT is mainly related to the activation of microglia^43^, over-activated microglia induce neuronal death and inhibit neuronal regeneration, leading to cognitive impairment in patients^43–45^. CD68 is a marker for activated microglia^46^. Dokic et al. found that CD68 signal in mouse brain increased 1.75-fold at 7 days after proton irradiation compared with the control samples (*p* < 0.05)^33^. The data of this study showed that the positive expression rate of CD68 in proton group was significantly higher than that in photon group, both at 20 Gy and 30 Gy (20 Gy: *p* < 0.001; 30 Gy: *p* = 0.022), suggesting that protons are more effective than photons in stimulating the activation and proliferation of microglia in the brain. As a result, the RBE of microglia was falls within the range of 1.1–1.65.

Radiation can also cause infiltration of large numbers of immune cells in brain tissue, which participate in the neuroinflammatory response in the brain. Long-term exposure leads to RIBI, which in turn causes hippocampal dysfunction^47^. CD45, a receptor-linked protein tyrosine phosphatase expressed on all immune cells^48^, was found to have similar expression levels between proton and photon irradiation (RBE = 1.1), implying that both modes of irradiation may have comparable effects on activating immune cells in the brain. This similarity is attributed to the disruption of the blood–brain barrier, allowing peripheral immune cells into the brain. The interaction of these immune cells is a dynamic and long-term process, necessitating an extended observation period to discern differences in how the two modes of irradiation affect immune cells.

## Conclusion

Our data confirm that protons are more powerful than photons in damaging neurons, promoting apoptosis, activating NSCs, inhibiting oligodendrocytes and activating microglia in the hippocampus, eventually leading to increased radiation toxicity. Therefore, the actual RBE of protons in the hippocampus must be higher than 1.1. It is suggested that we should pay more attention to protect hippocampus by control its dose of exposure as much as possible in clinical proton therapy. Further research is necessary to establish the ideal therapeutic dose and irradiation range for PBT in brain malignancies.

### Supplementary Information

Supplementary Information.

## Data Availability

The datasets generated during and/or analyzed during the current study are available from the corresponding author on reasonable request.
