# Supplementary material for: Evaluation of specific RBE in different cells of hippocampus under high-dose proton irradiation in rats
Source: Sci Rep. 2024 Apr 8;14:8193. doi: 10.1038/s41598-024-58831-z (PMC11001863; doi:10.1038/s41598-024-58831-z)

Title page

Evaluation of specific RBE in different cells of hippocampus under high-dose proton irradiation in rats

Running title: relative biological effectiveness and proton beam therapy;

Shengying Zhou[1, 2, #], Xingchen Ding[2, #], Yiyuan Zhang[2, #], Yuanyuan Liu[3], Xiaowen Wang[2,4], Yujiao Guo[5], Jianguang Zhang[6], Xiao Liu[7], Guanzhong Gong[2], Ya Su[2], Lizhen Wang[2], Miaoqing Zhao[3,*]and Man Hu[2, *]

[1] School of Clinical Medicine, Shandong Second Medical University, Weifang, 261053, Shandong, China

[2] Department of Radiation Oncology, Shandong Cancer Hospital and Institute, Shandong First Medical University and Shandong Academy of Medical Sciences, Jinan, 250117, Shandong, China

[3] Department of pathology, Shandong Cancer Hospital and Institute, Shandong First Medical University and Shandong Academy of Medical Sciences, Jinan, 250117, Shandong, China

[4] Shandong University, Jinan, 250100, Shandong, China

[5] Affiliated Hospital of Jining Medical College, Jining, 272067, Shandong, China

[6] Zibo wanjie cancer hospital, Zibo, 255202, Shandong, China

[7] 960 Hospital of the Joint Logistics Support Force of the Chinese People's Liberation Army, Jinan, 250031, Shandong, China

[#]These authors contributed equally to this work

[*]Correspondence authors (also responsible for statistical analyses):

Man Hu

Department of Radiation Oncology, Shandong Cancer Hospital and Institute, Shandong First Medical University and Shandong Academy of Medical Sciences, NO.440 Ji Yan Road, Jinan 250117, Shandong, China

Email: human5770@163.com

Phone: 86-0531-67626152

Fax: 86-0531-67626153

Miaoqing Zhao

Department of pathology, Shandong Cancer Hospital and Institute, Shandong First Medical University and Shandong Academy of Medical Sciences, Jinan, 250117, Shandong, China

Email: zhaomqsd@163.com

Phone: 86-0531-67626152

Fax: 86-0531-67626153

Supplementary Table S1 The relevant parameters of proton irradiation.

	parameter
Energy	230 MeV
LET	1.7 KeV/μm
Dose rate	2 Gy/min
SOBP	3 cm

Supplementary Figure S1 Percentage of neuronal damage in different subregions of the hippocampus. a. Percentage of neuronal damage in different subregions of the hippocampus at 24 hours after 20Gy irradiation. b. Percentage of neuronal damage in different subregions of the hippocampus at 24 hours after 30Gy irradiation. c. Percentage of neuronal damage in different subregions of the hippocampus at 7 days after 20Gy irradiation. d. Percentage of neuronal damage in different subregions of the hippocampus at 7 days after 30Gy irradiation.

Supplementary Fig. S1

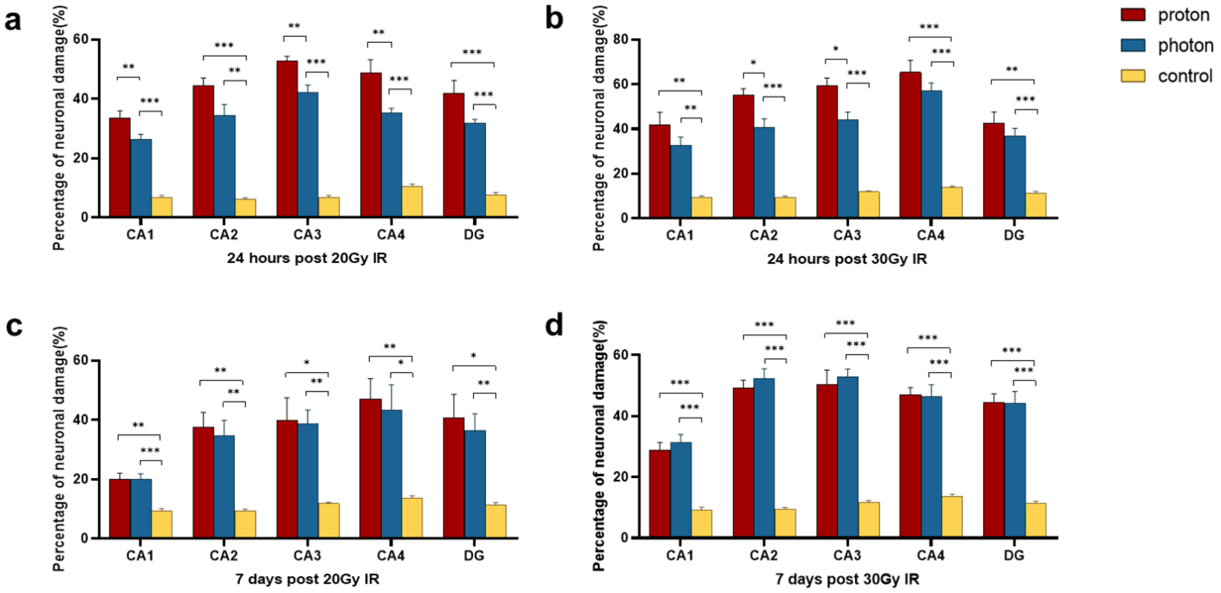

Supplement: Supplementary file 1 — Supplementary Information. [file 41598_2024_58831_MOESM1_ESM.pdf]
